# Two-Stage Gamified Digital Assessment for Autism Spectrum Disorder Screening and the Limits of Differentiating Social Communication Disorder in Children and Adolescents: Cross-Sectional Diagnostic Accuracy Study Using Explainable Machine Learning

**DOI:** 10.2196/102714

**Published:** 2026-09-23

**Authors:** Minyoung Jung, Ju Ran, Ennyoung Lee, Youngkyung Sunwoo, SooYeon Kim, Ji-Hoon Kim, Sungja Cho

**Affiliations:** 1 Department of Brain Convergence, Korea Brain Research Institute, 61 Cheomdan-ro, Dong-gu, Daegu, 41062, Republic of Korea, +82-53-980-8126; 2 Neudive Inc, Seoul, Republic of Korea; 3 Department of Psychiatry, Incheon Medical Center, Incheon, Republic of Korea; 4 Department of Psychiatry, Purme Foundation Nexon Children’s Rehabilitation Hospital, Seoul, Republic of Korea; 5 Department of Psychiatry, Pusan National University Yangsan Hospital, Yangsan, Republic of Korea

**Keywords:** autism spectrum disorder, social communication disorder, differential diagnosis, digital biomarker, gamification, machine learning, explainable artificial intelligence, nested cross-validation, feature-selection leakage, diagnostic screening

## Abstract

**Background:**

Distinguishing autism spectrum disorder (ASD) from social communication disorder (SCD) is clinically challenging because both conditions present with overlapping social communication deficits. Standard caregiver-reported instruments capture surface-level behavioral similarities rather than underlying cognitive differences, motivating the development of digital gamified assessments that measure social cognitive processes directly.

**Objective:**

This study developed and evaluated a 2-stage gamified digital pipeline: stage 1 (Buddy Plan, a self-report module) for high-sensitivity ASD screening, and stage 2 (Buddy Drill, story-based social-judgment scenarios), which was examined with a leakage-controlled analysis, for assessing whether ASD can be differentiated from SCD.

**Methods:**

In this cross-sectional diagnostic accuracy study, 275 children and adolescents aged 6‐18 years (mean 11.07, SD 3.13 years; 175/275, 63.6% male) were recruited by convenience sampling from 5 clinical and community sites in the Republic of Korea (May 2024 to February 2025) across 5 Diagnostic and Statistical Manual of Mental Disorders, Fifth Edition (DSM-5) groups: ASD (n=51), SCD (n=54), attention-deficit/hyperactivity disorder (n=23), high risk (n=52), and neurotypically developing (ND; n=95). Diagnoses were established by board-certified child psychiatrists. Participants completed 2 tablet-based modules: Buddy Plan (52 self-report items; stage 1) and Buddy Drill (153 story-based scenarios; stage 2). The primary outcome was diagnostic accuracy (area under the receiver operating characteristic curve [AUC], sensitivity, and specificity). Four machine learning algorithms were trained with nested cross-validation (5×5 folds). For stage 2, item selection and imputation were performed within each training fold. Explainability used Shapley Additive Explanations (SHAP). Significance was set at α=.05 (2-sided) with bootstrap 95% CIs.

**Results:**

Group differences were tested by 1-way ANOVA. For stage 1 (ASD vs ND; n=146), random forest achieved a nested AUC of 0.912 (95% CI 0.856‐0.953). At a threshold of 0.200, sensitivity was 96.1% (49/51; 95% CI 86.8%‐99.5%) and specificity was 58.9% (56/95; 95% CI 48.4%‐68.9%), with 2 false negatives. For stage 2 (ASD vs SCD; n=100), the fully nested pipeline yielded only chance-level discrimination: regularized logistic regression achieved a nested AUC of 0.62 (95% CI 0.51‐0.74), and no feature configuration (self-report: 0.55, objective: 0.62, combined: 0.63) exceeded chance. SHAP identified 5 cross-algorithm stage 1 biomarkers with significant ASD-versus-ND differences (all *P*<.01) and no evidence of sex bias.

**Conclusions:**

The gamified Buddy Plan module shows promise for high-sensitivity ASD screening. In contrast, once feature-selection leakage was removed with a fully nested pipeline, the Buddy Drill module did not robustly differentiate ASD from SCD, and the apparent advantage of objective features over self-report features seen in leaky analyses did not persist. Because the results derive from internal cross-validation in a single, predominantly male Korean cohort without external validation or IQ matching, they represent preliminary evidence of screening feasibility rather than validated clinical differentiation. Prospective, externally validated, IQ- and language-matched studies are required.

## Introduction

### Background

Autism spectrum disorder (ASD) affects approximately 1 in 36 children in the United States, with diagnosis frequently delayed beyond the age of 4‐5 years despite the established benefits of early intervention [1,2]. A particularly salient clinical challenge lies in differentiating ASD from social communication disorder (SCD)—conditions that share core impairments in pragmatic communication, social reciprocity, and nonverbal communication but differ primarily in the presence of restricted and repetitive behaviors (RRBs) [3]. This diagnostic boundary has profound consequences: ASD and SCD require different intervention strategies, prognostic counseling, and service eligibility pathways, yet clinicians struggle to reliably distinguish these conditions [4]. The challenge is compounded by the fact that SCD was introduced in the Diagnostic and Statistical Manual of Mental Disorders, Fifth Edition (DSM-5) specifically to classify children with social communication deficits who lack the RRBs characteristic of ASD. However, high-functioning children with ASD whose RRBs are subtle or context-dependent may be misclassified as having SCD, leading to inappropriate intervention planning and delayed access to ASD-specific services.

Existing gold-standard instruments, such as the Social Responsiveness Scale-2 (SRS-2) and Vineland Adaptive Behavior Scales (VABS), rely on parent or caregiver report [5,6]. These instruments measure observable social behavior through a third-party lens that captures what an informant perceives about the child’s social functioning. While psychometrically validated for broad screening, they assess surface-level behavioral phenotypes that appear similar in ASD and SCD [7]. When an observer rates a child’s difficulty with understanding social cues, the outward manifestation may be identical in both conditions even though the underlying cognitive mechanisms differ.

Digital health technologies provide a mechanism for the direct assessment of internal social cognitive processes [8,9]. Gamified applications can embed psychometric measurement within engaging, narrative-driven interactive experiences [10]. Story-based social judgment tasks requiring interpretation of social cues, theory of mind (ToM) reasoning, and behavioral outcome prediction engage the same cognitive processes that are differentially impaired in ASD versus SCD [11,12]. Unlike observer ratings that capture behavioral endpoints, these performance-based tasks access the social information processing system, potentially revealing qualitative differences between conditions. Specifically, ToM deficits in ASD are thought to reflect an impairment in the ability to represent and reason about others’ mental states, whereas SCD is characterized by more circumscribed difficulties in the pragmatic application of social communication skills with relatively preserved ToM capacity [11,12]. A digital assessment capable of probing these differential cognitive mechanisms could therefore provide the discriminative power that observer-rated instruments lack. In addition, a clinically practical screening tool must address 2 distinct questions sequentially: (1) “Does this child have clinically significant social communication difficulties?” and (2) “If so, is the pattern more consistent with ASD or SCD?” This sequential logic demands a 2-stage pipeline with different optimization targets at each stage.

However, translating digital behavioral data into reliable clinical decision support requires methodological transparency. The Consolidated Reporting Guidelines for Machine Learning Modeling Studies (CREMLS) emphasize multialgorithm comparison, calibration assessment, and transparent feature selection [13]. Machine learning (ML) approaches to ASD screening have shown promise, with several studies achieving area under the receiver operating characteristic curve (AUC) values above 0.85 for differentiating individuals with ASD from typically developing controls [14-16]. However, critical gaps persist. Despite recent advances, multialgorithm digital screening tools rarely quantify error propagation across sequential diagnostic stages. Furthermore, the comparative utility of subjective behavioral ratings versus objective, performance-based tasks for ASD-SCD differentiation remains underexplored within a unified digital platform. Existing models also frequently lack granular explainability, such as Shapley Additive Explanations (SHAP)–based feature attribution, across all stages of a clinical pipeline and often fail to rigorously control for demographic confounds like sex bias—a concern given that the male-skewed ASD prevalence is inadequately controlled in most studies [17].

This study aimed to address these gaps by developing and evaluating a 2-stage digital assessment pipeline using a gamified application with 2 complementary modules. For stage 1 (broad screening), we developed Buddy Plan, a gamified self-report module that captures subjective social competence ratings across multiple domains of social-communicative functioning. For stage 2 (differential diagnosis), we developed Buddy Drill, an objective story-based social judgment module grounded in ToM and social information processing frameworks, designed to probe the internal cognitive processes that may differentially distinguish ASD from SCD. To ensure methodological rigor and transparency, all classification analyses used nested cross-validation (5×5 stratified folds) for unbiased performance estimation; multialgorithm benchmarking across 4 ML classifiers (random forest [RF], support vector machine [SVM], gradient boosting [GB], and logistic regression [LR]); correlation-based, item response theory (IRT)–inspired psychometric item selection for stage 2 feature refinement; complete SHAP analysis for cross-algorithm explainability; calibration assessment via Brier scores; and sex-stratified sensitivity testing to evaluate potential demographic bias.

## Methods

### Study Design and Participants

This cross-sectional study enrolled 275 children aged 6‐18 years (mean 11.07, SD 3.13 years; 175/275, 63.6% male) between May 2024 and February 2025 across 5 diagnostic groups: ASD (n=51), SCD (n=54), attention-deficit/hyperactivity disorder (ADHD; n=23), high risk (HR; n=52), and neurotypically developing (ND; n=95). Participants were recruited from 5 sites: Purme Foundation Nexon Children’s Rehabilitation Hospital (Seoul), Incheon Medical Center (Incheon), Ewha Womans University Medical Center (Seoul), Pusan National University Yangsan Hospital, and Korea Brain Research Institute (Daegu). Clinical diagnoses followed DSM-5 criteria established by board-certified child psychiatrists (3 senior clinicians across 3 clinical recruitment sites). For each participant, the diagnosing clinician conducted a structured clinical interview with parents and direct behavioral observation of the child. Cases with an uncertain diagnosis were discussed in a multidisciplinary consensus conference attended by at least 2 clinicians; consensus was required before enrollment.

This was a cross-sectional, single-wave diagnostic accuracy study conducted and reported in accordance with the Journal Article Reporting Standards (JARS) for quantitative research and the guidelines for developing and reporting machine-learning predictive models in biomedical research [18].

### Setting

Recruitment took place across 5 clinical and community sites in the Republic of Korea (3 hospital-based child psychiatry clinics, 1 university medical center, and 1 research institute) between May 2024 and February 2025.

### Inclusion and Exclusion Criteria

The inclusion criteria were age 6-18 years; sufficient ability to complete a tablet-based assessment; and, for the clinical groups, a DSM-5 diagnosis confirmed by a board-certified child psychiatrist. The exclusion criteria were an uncorrected sensory or motor impairment precluding tablet use and inability to provide assent.

### Sampling Procedures

Participants were enrolled using convenience (consecutive clinical-referral and community-volunteer) sampling; no probability-based sampling frame was used, which is acknowledged as a potential source of selection bias in the Limitations section.

### Sample Size, Power, and Precision

An a priori power analysis was not performed because the sample comprised all eligible participants accrued during the fixed recruitment window. Accordingly, precision is conveyed by bootstrap 95% CIs throughout, and the study is framed as providing preliminary, internally validated estimates rather than definitive effect sizes.

### Measures and Covariates

The primary measures were the Buddy Plan and Buddy Drill digital modules, which are described below. Covariates comprised age; sex; and, where available, full-scale IQ (FSIQ), SRS-2, and VABS. A JARS-style participant flow diagram summarizing enrollment, module administration, and analytic inclusion at each stage is provided in Multimedia Appendix 1.

### Ethical Considerations

The study protocol was reviewed and approved by the Institutional Review Boards (IRBs) of the following 4 institutions: Public Institutional Bioethics Committee (approval number: P01-202502-01-028), Incheon Medical Center (approval number: 115288‐202408 HR-092-06), Ewha Womans University (approval number: ewha-202501-0001-02), and Pusan National University Yangsan Hospital (approval number: 10-2024-014). Written informed consent was obtained from all parents or legal guardians, and written assent was obtained from all child participants aged 7 years or older, in accordance with institutional policy. Participants received no financial compensation for study participation. All personally identifiable information was removed prior to analysis, and data were stored on encrypted, access-controlled institutional servers. All procedures were conducted in accordance with the Declaration of Helsinki. Children could withdraw from the assessment at any time without consequence, and research staff monitored for signs of fatigue or distress throughout the gamified assessment sessions. No individual participant is identifiable in any image, figure, or supplementary material in this manuscript. All figures present aggregated, deidentified data or schematic illustrations only, and no photographs or other potentially identifying images of participants are included.

### Digital Assessment Platform

**Figure 1.**
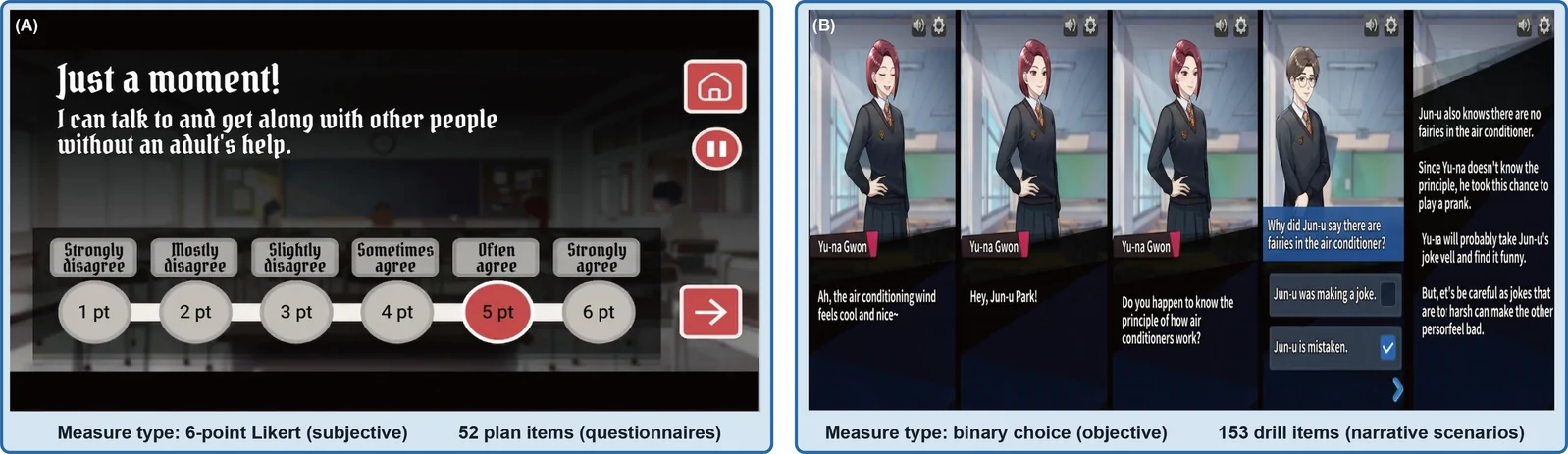
Overview of the 2-module gamified digital assessment platform. (A) Buddy Plan (social competence rating module): a gamified self-report questionnaire comprising 52 items (P-items) in which the child rates social behaviors on a 6-point Likert scale. (B) Buddy Drill (story-based social judgment module): an objective, narrative-driven interactive assessment comprising 153 scenarios (D-items) grounded in theory of mind and social information processing frameworks. Each scenario presents a social situation requiring the child to interpret implicit social cues, engage in perspective-taking, and predict appropriate behavioral outcomes through a binary response (correct/incorrect).

The gamified digital assessment was administered via tablet devices and consisted of 2 primary modules: Buddy Plan and Buddy Drill (Figure 1). The digital assessment modules, Buddy Plan and Buddy Drill, were developed by operationalizing core diagnostic features from established clinical instruments, including SRS-2 and VABS. To ensure a comprehensive digital phenotype, the content was mapped onto four critical domains of social-functional impairment: (1) situational awareness (recognition of social cues and environmental context), (2) communication (pragmatic language use and nonverbal communicative intent), (3) social motivation (the drive to initiate and maintain social engagement), and (4) restricted interests and behaviors (identification of rigid processing patterns, particularly in social reasoning scenarios).

### Buddy Plan (Social Competence Rating Module)

The Buddy Plan module comprised 52 items (P-items) evaluating social behaviors, including social awareness, communicative intent, empathy, and social motivation. Items were presented as gamified self-report questions completed directly by the child participant on a tablet device, with a 6-point Likert response scale ranging from 1 (never/not like me) to 6 (always/very much like me). Reading-level appropriateness was ensured by keeping all items at a grade 3 reading level or below. A trained research assistant was present to provide item clarification for younger participants (aged 6‐8 years) if requested. This child self-report approach distinguishes Buddy Plan from parent-informant instruments such as SRS-2 and VABS, enabling direct capture of the child’s subjective social experience rather than observer perception. However, self-report reliability is known to vary with age, cognitive ability, and insight capacity; younger children (6‐8 years) may have limited metacognitive awareness of their own social difficulties, while adolescents may demonstrate response bias related to social desirability [19]. Our study did not collect IQ or language proficiency data, which are potential confounders for self-report validity in this population. To ensure unidirectional interpretation—where higher scores consistently reflect greater social difficulty—20 of the 52 items were reverse-scored (calculated as 7 minus the original score). This module served as the primary feature set for stage 1 (broad screening). To provide a theoretically grounded interpretation of the ML results and facilitate clinically meaningful profiling across diagnostic groups, the 52 Buddy Plan items were organized into 5 theoretically derived subscales based on expert consensus classification. A panel of board-certified child psychiatrists and developmental psychologists independently categorized each item based on its target construct, drawing on established frameworks from the social cognition, social communication, and neurodevelopmental literature [3,4,11,12]. Items were assigned to subscales through iterative discussion until unanimous agreement was reached. This classification is theoretically motivated rather than empirically derived; no exploratory factor analysis or confirmatory factor analysis was performed to validate the 5-factor structure statistically. Accordingly, the subscale structure should be interpreted as a clinically informed organizational framework, and the factor solution may differ from data-driven approaches. Further details on the resulting 5-factor structure, which captures complementary dimensions of social-communicative functioning, are described in Multimedia Appendix 2.

### Buddy Drill (Story-Based Social Judgment Module)

The Buddy Drill module was an objective, gamified assessment consisting of 153 narrative scenarios (D-items). Grounded in ToM and social information processing frameworks [11,20], this interactive story game evaluated the child’s ability to interpret implicit social cues, engage in perspective-taking, and predict appropriate behavioral outcomes. Each scenario concluded with a binary choice (correct or incorrect), yielding an objective performance metric free from informant bias. This module served as the primary feature set for stage 2 (differential diagnosis). The Buddy Drill module was administered exclusively to children in clinical and HR groups (ASD, SCD, ADHD, and HR). Due to the sequential gatekeeping design, stage 2 focused exclusively on differentiating between clinical phenotypes within the social-communication deficit spectrum, where children with ND would have already been filtered out at stage 1. This design decision also reflected practical considerations: (1) the IRB mandated minimization of assessment burden for participants with ND, as the 153-scenario module requires 40‐60 additional minutes of testing, and (2) there was an anticipated ceiling effect in children with ND, inferred indirectly from the HR group’s mean accuracy (D_mean=0.771). The HR group, while the least clinically affected group that received D-items, does not represent children with ND, and this inference therefore serves as a practical justification rather than an empirical demonstration. Future studies should administer Buddy Drill to participants with ND to establish full pipeline validity across all diagnostic groups.

### Statistical Analyses

Missing data were minimal and are reported explicitly here. No participant had more than 5% missing Buddy Plan (P-item) responses, and overall P-item missingness was below 2% of all item responses. These few missing values were resolved by within-fold median imputation (applied within each cross-validation training fold to prevent data leakage), and with a missing fraction this small, multiple imputation was not expected to materially alter estimates and was therefore not used. Across the clinical covariates that contained sporadic missingness (SRS-2 and VABS subscales; 0.7% of values), the Little missing completely at random (MCAR) test was nonsignificant (*χ*²_9_=5.9; *P*=.75), consistent with a MCAR mechanism. In contrast, Buddy Drill (D-item) and FSIQ data were missing by design rather than at random—the Buddy Drill module was not administered to the ND group (see the Buddy Drill section) and FSIQ was not collected for all participants—and this missingness was strongly nonrandom (Little MCAR test: *χ*²_9_=195.1; *P*<.001). Accordingly, D-item analyses were restricted to participants who completed the module, and no D-item imputation was performed. No outlier exclusions were applied, as all responses fell within the valid Likert range (1-6). Class imbalance between ASD (n=51) and ND (n=95) was addressed through class-weighted algorithms (class weight=“balanced” for SVM and LR; balanced class weights for RF and GB) rather than synthetic oversampling, preserving the original multidimensional clinical phenotype distributions, which synthetic oversampling methods may distort in high-dimensional feature spaces. The 5×5 nested cross-validation design was selected to balance the bias-variance tradeoff in performance estimation: 5 outer folds provided sufficient held-out data per fold (approximately 29 samples), while 5 inner folds enabled robust hyperparameter selection within each training partition. To prevent optimistic bias from information leakage between tuning and evaluation, all models used nested cross-validation [21] (5×5 stratified folds). The outer loop provided unbiased generalization estimates on held-out data never used for hyperparameter selection, while the inner loop conducted exhaustive grid search (GridSearchCV) over predefined hyperparameter spaces. Feature standardization was applied within each outer training fold for algorithms requiring it (LR and SVM), preventing data leakage. Four algorithms were benchmarked: RF (324 combinations), GB (288 combinations), SVM (40 combinations), and LR (12 combinations). To ensure transparency in the classification models, we used SHAP [22] to quantify the contribution of each digital biomarker to individual predictions. For tree-based models (RF and GB), exact TreeSHAP was applied, computing feature-level contributions in polynomial time. For SVM, KernelSHAP with k-means summarization (k=50) of the background distribution was used. Both global importance (mean |SHAP|) and directional effects (beeswarm plots) were examined across all algorithms. Consensus biomarkers were defined as features ranking in the top 10 by SHAP across 3 or more algorithms. Given the unbalanced sex distribution (ASD: 43/51, 84% male; ND: 49/95, 52% male), the following three analyses tested for sex bias: (1) male-only subgroup analysis, (2) sex-inclusive models with sex as an additional (53rd) feature, and (3) SHAP-based quantification of sex’s contribution to predictions. Differential item functioning (DIF) analysis by sex was not performed within the item selection framework owing to insufficient sample sizes within sex-by-diagnosis subgroups (female ASD: n=8; female SCD: n=6). DIF analysis is a priority for future studies with larger, sex-balanced cohorts to ensure that individual Buddy Plan and Buddy Drill items do not exhibit measurement bias by sex. An exploratory age-stratified analysis was conducted by dividing participants into younger (6‐11 years; n=171) and older (12‐18 years; n=104) subgroups. Stage 1 RF performance remained robust in both subgroups (younger AUC=0.896; older AUC=0.921), though the small subgroup sample sizes preclude definitive conclusions about age-specific performance. The age range of 6‐18 years represents a substantial developmental span; age-normed scoring (adjusting item difficulty or subscale interpretation by developmental stage) was not implemented in our study but is recommended for future instrument development to account for maturational differences in social cognitive capacity across childhood and adolescence. Model calibration was assessed via calibration curves and Brier scores. Digital P-items were benchmarked against demographics only (age and sex) and a clinical gold standard (age, sex, SRS-2, and VABS). Cross-group generalization analysis applied the ASD-versus-ND–trained model to all 5 groups to assess dimensional sensitivity.

### Principal Component Analysis–Based Dimensionality Reduction (Neurodevelopmental Scores)

To reduce potential redundancy across correlated behavioral features and extract latent cognitive phenotypes from the gamified assessments, we applied a principal component analysis (PCA)–based dimensionality reduction framework adapted from the E-score methodology described by Park et al [23]. This approach reduces redundancy across behavioral features by grouping correlated items and extracting composite scores that capture underlying neurodevelopmental dimensions. First, all 52 P-item scores were standardized into *z* scores, and outliers exceeding |2.5| SDs were capped at ±2.5 to prevent outlier dominance in PCA [23]. A global PCA was then applied to the feature correlation matrix to project all items onto a 2D principal component space. K-means clustering (k=3; selected via the elbow method) was applied to this 2D space to identify groups of items with similar covariance structures. Finally, within each cluster, a cluster-specific PCA was conducted, and the first principal component score (PC1) was extracted for each participant, yielding 3 composite “N-scores” (neurodevelopmental scores). These N-scores were evaluated for clinical validity through partial correlation analyses controlling for age and through group comparison analyses (ASD vs ND for P-item N-scores; ASD vs SCD for D-item N-scores). The classification performance of N-scores was compared against raw item scores using LR and RF with 10×5 repeated stratified cross-validation. A hybrid analysis further combined correlation-based selected D-items with P-item N-scores. Because the PCA-based N-score derivation and the item selection for these secondary analyses were performed on the full sample rather than within cross-validation folds, they are exploratory and, like the original stage 2 estimate, are subject to optimistic bias. They are not used to support the study’s conclusions. The complete mathematical specification of this 5-step pipeline (equations 1-5)—including item-level *z*-score standardization, ±2.5 SD winsorization, eigendecomposition of the 52×52-item correlation matrix, k-means partitioning in the 2D loading space, and cluster-specific PC1 extraction—is provided in Multimedia Appendix 3.

### Stage 1: Buddy Plan Screening (ASD vs ND)

Stage 1 classified ASD versus ND using all 52 P-items. Because stage 1 functions as a screening gate, where any false negative represents an irrecoverable error propagating through the pipeline, we optimized the classification threshold for ≥95% sensitivity, accepting reduced specificity to minimize missed ASD cases. The threshold was independently optimized within each outer fold’s training partition and applied exclusively to that fold’s held-out test data, ensuring no information leakage from cross-fold threshold selection. The reported sensitivity (96.1%) and specificity (58.9%) at a threshold of 0.200 reflect aggregated outer-fold performance at this threshold value. Error propagation was quantified by applying the trained stage 1 model to all 5 diagnostic groups (ASD, SCD, ADHD, HR, and ND) as a cross-group generalization analysis of dimensional sensitivity. The ADHD group (n=23) was not used in any primary classification analysis. It served exclusively in this cross-group generalization analysis to assess whether the ASD screening model appropriately differentiated ASD from a clinically relevant comparison group with overlapping behavioral features.

### PCA-Based N-Score Analysis and Information Dilution Resolution

PCA-based clustering of the 52 P-items yielded 3 item clusters (19, 26, and 7 items; cumulative variance explained by PC1 and PC2: 78.8%), from which 3 N-scores were extracted. The second N-score (P_N2), derived from a 26-item cluster, demonstrated the largest effect size for ASD versus ND differentiation (Cohen *d*=2.03; *t*_144_=12.04; *P*<.001). This large effect exceeds those observed with raw total scores (Cohen *d*=1.41 for P_sum), representing a 44% improvement in effect size through dimensionality reduction. P_N2 showed strong partial correlations (controlling for age) with SRS-2 total (*r*=0.458; *P*<.001), SRS-2 RRB (*r*=0.602; *P*<.001), and VABS-ABC (*r*=–0.552; *P*<.001), suggesting it captures a core social difficulty dimension with particular sensitivity to RRB features. Using only 3 N-scores, LR achieved an AUC of 0.910 for ASD versus ND classification—exceeding the 52-item LR AUC of 0.881 despite using 94% fewer features. Because this PCA-based extraction was performed on the full sample rather than within cross-validation folds, it is reported as an exploratory dimensionality-reduction result rather than a leakage-free performance estimate.

For ASD versus SCD differentiation, an exploratory hybrid approach combining selected D-items with P-item N-scores was also examined. Because both the N-score derivation and the D-item selection for this analysis were performed on the full sample, the resulting estimates are subject to the same feature-selection leakage identified for the primary stage 2 analysis and are reported only as exploratory. They should not be interpreted as leakage-free evidence of ASD-SCD differentiation.

### Stage 2: Buddy Drill Differential Diagnosis (ASD vs SCD)

A correlation-based item selection procedure, inspired by IRT principles, identified maximally discriminating Buddy Drill scenarios. Because the sample size (n=100) was insufficient for formal IRT modeling via marginal maximum likelihood estimation—which typically requires 200‐500 respondents per parameter for stable 2PL estimates [24]—a simplified approximation was used: the discrimination parameter *(a)* was estimated via point-biserial correlation with logistic scaling (×1.7), and the difficulty parameter *(b)* was estimated via probit transformation of accuracy. Unidimensionality was supported by a PC1/PC2 eigenvalue ratio of 2.33 (threshold: 2.0), though this is less conservative than the commonly cited 3.0 threshold, and 11.1% of item pairs exceeded the local independence criterion of |r|=0.20. Items were ranked by discrimination, and the top 50 were selected (IRT-50 set). An abbreviated 5-item set (IRT-5) was also evaluated for rapid screening feasibility. We acknowledge that this approach does not constitute formal IRT analysis; accordingly, we refer to it as “correlation-based item selection inspired by IRT” throughout this manuscript. In response to peer review and to eliminate feature-selection leakage, the entire item-ranking, selection, and median-imputation procedure was recomputed independently within each outer training fold of the nested cross-validation (a fully nested pipeline). The leakage-free estimates from this pipeline are those reported for stage 2. Estimates from the earlier version, in which selection was performed once on the full ASD-SCD sample, are identified as such and are not used to support the study’s conclusions.

## Results

### Participant Characteristics

A total of 275 children aged 6-18 years (mean age 11.07, SD 3.13 years; 175/275, 63.6% male) were enrolled across 5 diagnostic groups (Table 1). One-way ANOVA revealed significant group differences across all continuous variables (all *P*<.001). The ASD group (n=51; mean age 12.00, SD 3.49 years; 43/51, 84% male) demonstrated the highest SRS-2 scores (mean 82.86, SD 15.38) and lowest VABS composite scores (mean 64.20, SD 11.60), indicating the most severe social communication impairment and adaptive functioning deficits. The SCD group (n=54; mean age 9.94, SD 2.84 years; 48/54, 89% male) showed moderately elevated SRS-2 scores (mean 72.47, SD 13.80) and low VABS scores (mean 68.13, SD 6.87). The ADHD group (n=23; mean age 10.00, SD 2.13 years; 18/23, 78% male) had a mean SRS-2 score of 67.64 (SD 18.15) and a mean VABS score of 70.32 (SD 8.63). The HR group (n=52; mean age 13.98, SD 2.66 years; 17/52, 33% male) had a mean SRS-2 score of 75.54 (SD 19.59) and a mean VABS score of 79.71 (SD 18.50). The ND group (n=95; mean age 9.86, SD 2.24 years; 49/95, 52% male) had the lowest SRS-2 scores (mean 41.97, SD 33.75) and highest VABS scores (mean 108.89, SD 16.50). Sex distribution differed significantly across groups (*χ*^2^=53.92; *P*<.001; V=0.443), driven primarily by the lower male proportion in the HR group (17/52, 33%). On the Buddy Plan digital assessment, the ASD group reported the greatest overall social difficulty (P_mean=3.944, SD 0.478), followed by the HR group (P_mean=3.862, SD 0.569), SCD group (P_mean=3.772, SD 0.450), and ADHD group (P_mean=3.765, SD 0.351), with the ND group reporting the least difficulty (P_mean=3.378, SD 0.304; *F*_4,270_=19.63; *P*<.001; η^2^=0.225). Across the Buddy Plan subscales, the largest group effect was observed for total score (*F*_4,270_=33.04; *P*<.001; η^2^=0.329), followed by F1 (social cognition and contextual understanding; *F*_4,270_=30.11; *P*<.001; η^2^=0.308) and F4 (repetitive/restricted interests and sensory rigidity; *F*_4,270_=22.87; *P*<.001; η^2^=0.253). The ASD-ND difference was the largest for F4 (delta=+1.391) and F1 (delta=+1.250), consistent with the DSM-5 diagnostic criteria. Of particular diagnostic relevance, the ASD-SCD comparison revealed near-zero differences in F2 (interaction skills and nonverbal communication; delta=−0.007) and F5 (self-expression and self-regulation; delta=−0.013), confirming that these 2 conditions present identically on observable interaction behaviors. The largest ASD-SCD difference was observed in F4 (repetitive/restricted interests and sensory rigidity; delta=+0.266), consistent with the DSM-5 criterion that RRBs primarily distinguish ASD from SCD. Convergent validity was supported by significant correlations between P_mean and SRS-2 (*r*=0.290; *P*<.001) and between P_mean and VABS (*r*=−0.376; *P*<.001). Regarding internal consistency, while most subscales showed acceptable to good reliability (Cronbach α range: 0.666-0.871), F5 (self-expression and self-regulation) demonstrated lower internal consistency (α=.508), below the conventional acceptability threshold. A post hoc sensitivity analysis excluding all F5 items from the stage 1 feature set yielded negligible performance change (RF AUC=0.905 vs 0.912 with full items), confirming the model’s robustness to this subscale’s variance (Table S1 in Multimedia Appendix 2).

**Table 1.** Demographic, clinical, and Buddy Plan subscale characteristics by diagnostic group (N=275).

Variable	ASD^a^ (n=51)	SCD^b^ (n=54)	ADHD^c^ (n=23)	HR^d^ (n=52)	ND^e^ (n=95)	*F* test (*df*)	Chi-square (*df*)	*P* value	η²^f^	V^g^
Demographics										
Age, mean (SD)	12.00 (3.49)	9.94 (2.84)	10.00 (2.13)	13.98 (2.66)	9.86 (2.24)	24.64 (4,270)	—^h^	<.001	0.267	—
Male, n (%)	43 (84)	48 (89)	18 (78)	17 (33)	49 (52)	—	53.92 (4)	<.001	—	0.443
Clinical measures										
SRS-2^i^, mean (SD)	82.86 (15.38)	72.47 (13.80)	67.64 (18.15)	75.54 (19.59)	41.97 (33.75)	97.31 (4,270)	—	<.001	0.590	—
VABS^j^, mean (SD)	64.20 (11.60)	68.13 (6.87)	70.32 (8.63)	79.71 (18.50)	108.89 (16.50)	179.76 (4,270)	—	<.001	0.727	—
Buddy Plan										
P_mean (SD)	3.944 (0.478)	3.772 (0.450)	3.765 (0.351)	3.862 (0.569)	3.378 (0.304)	19.63 (4,270)	—	<.001	0.225	—
Buddy Plan subscales^k^										
F1: Social cognition^l^	3.817 (0.763)	3.596 (0.858)	3.442 (0.639)	3.399 (0.901)	2.567 (0.622)	30.11 (4,270)	—	<.001	0.308	—
F2: Interaction skills^m^	2.971 (0.827)	2.978 (0.882)	2.731 (0.591)	2.453 (0.965)	2.300 (0.683)	9.54 (4,270)	—	<.001	0.124	—
F3: Motivation/anxiety^n^	3.453 (0.868)	3.263 (0.701)	2.787 (0.657)	3.577 (0.930)	2.554 (0.662)	21.29 (4,270)	—	<.001	0.240	—
F4: RRB^o^/sensory^p^	4.072 (1.067)	3.806 (0.986)	3.486 (0.992)	3.615 (1.120)	2.681 (0.749)	22.87 (4,270)	—	<.001	0.253	—
F5: Self-expression^q^	3.146 (0.833)	3.159 (0.741)	3.211 (0.883)	3.266 (0.866)	2.605 (0.645)	9.36 (4,270)	—	<.001	0.122	—
Total	3.514 (0.613)	3.373 (0.609)	3.152 (0.466)	3.237 (0.696)	2.527 (0.497)	33.04 (4,270)	—	<.001	0.329	—

a ASD: autism spectrum disorder.

b SCD: social communication disorder.

c ADHD: attention-deficit/hyperactivity disorder.

d HR: high risk.

e ND: neurotypically developing.

f η²: eta-squared (proportion of variance explained).

g V: Cramér V.

h Not applicable.

i SRS-2: Social Responsiveness Scale-2.

j VABS: Vineland Adaptive Behavior Scales.

k Higher Buddy Plan scores reflect greater social difficulty.

l F1: Social cognition and contextual understanding.

m F2: Interaction skills and nonverbal communication.

n F3: Social motivation and avoidance/anxiety.

o RRB: restricted and repetitive behavior.

p F4: Repetitive/restricted interests and sensory rigidity.

q F5: Self-expression and self-regulation.

### Stage 1: Buddy Plan Screening

RF achieved the highest nested AUC of 0.912 (SD 0.054) with a precision-recall AUC of 0.869, *F*_1_-score of 0.744, sensitivity of 0.627, and specificity of 0.968. SVM with a linear kernel yielded the second highest AUC of 0.885 (SD 0.097). GB achieved an AUC of 0.869 (SD 0.066) with a comparable *F*_1_-score and specificity. LR had the lowest AUC of 0.826 (SD 0.093). All reported values reflect outer-fold predictions that were never used for hyperparameter tuning.

**Figure 2.**
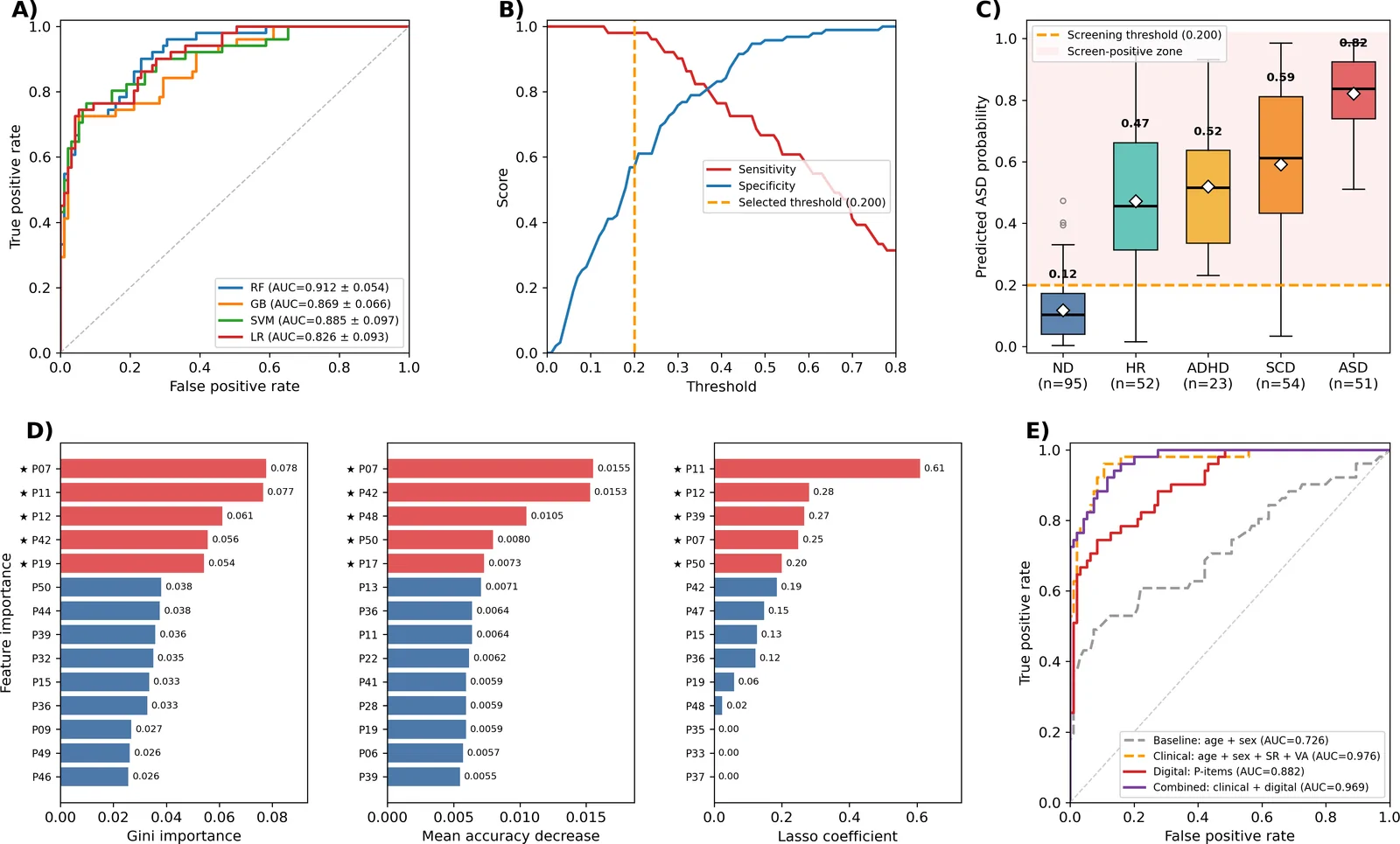
Stage 1 screening performance and threshold optimization for autism spectrum disorder (ASD) versus neurotypically developing (ND) classification (n=146). (A) Receiver operating characteristic (ROC) curves for 4 machine learning algorithms (random forest [RF], support vector machine [SVM], gradient boosting [GB], and logistic regression [LR]) with nested cross-validation area under the curve (AUC) values. RF achieved the highest AUC of 0.912 (SD 0.054). (B) Sensitivity-specificity tradeoff as a function of classification threshold, illustrating the optimized threshold of 0.200 that achieved 96% sensitivity (49/51) with 2 ASD false negatives (2/51). (C) Box plot of AUC distributions across algorithms comparing digital P-items, demographics-only baseline, and clinical gold standard models. (D) Feature importance rankings derived from Gini importance (RF), permutation importance (RF), and linear coefficients (LR). (E) ROC curves comparing feature set configurations: demographics only (AUC=0.726), digital P-items alone (AUC=0.912), clinical gold standard with Social Responsiveness Scale-2 and Vineland Adaptive Behavior Scales (AUC=0.976), and combined clinical plus digital features (AUC=0.969). ADHD: attention-deficit/hyperactivity disorder; HR: high risk; SCD: social communication disorder.

RF achieved the highest nested AUC (0.912, SD 0.054) with excellent specificity (0.968) at the default threshold of 0.500. However, sensitivity at the default threshold was 0.627, insufficient for a screening tool. We optimized the threshold to 0.200, yielding 96.1% sensitivity with 2 ASD false negatives (2/51) (Figure 2A and B). Model calibration assessment revealed that the RF model achieved a Brier score of 0.132 (stage 1), indicating good probabilistic accuracy. The calibration curve showed slight overconfidence in the mid-range probability zone (predicted probabilities of 0.3‐0.6 were approximately 5‐10 percentage points higher than observed frequencies), which is common in tree-based classifiers. For stage 2, the LR model achieved a Brier score of 0.207, reflecting moderate calibration consistent with the more challenging ASD-SCD discrimination. The stage 2 calibration curve demonstrated better calibration than stage 1 for the LR model, as LR inherently produces well-calibrated probabilities through its sigmoidal output function. For practical use, these calibration findings imply that if the model output is to serve as a risk score, mid-range stage 1 probabilities should be interpreted with mild caution given the observed overconfidence, and recalibration on the intended deployment population (eg, Platt scaling or isotonic regression) is advisable before threshold-based clinical decisions are made. Without any clinician involvement, digital P-items achieved a nested AUC of 0.912—an 18.6 percentage-point improvement over demographics alone (AUC=0.726) and 93% of the clinical gold-standard performance (AUC=0.976). The clinical gold standard required trained administrators to collect SRS-2 and VABS scores; the digital tool required none. The marginal degradation when combining clinical and digital features (0.976-0.969) reflected expected collinearity in a small sample with near-ceiling clinical performance. As illustrated in the receiver operating characteristic curves (Figure 2A), all 4 algorithms achieved AUC values exceeding 0.82, with RF demonstrating the most robust discrimination. The sensitivity-specificity tradeoff curve (Figure 2B) revealed that lowering the classification threshold from 0.500 to 0.200 shifted the operating point from high specificity (0.968) and low sensitivity (0.627) to the clinically preferred configuration of 96.1% sensitivity and 58.9% specificity, ensuring that virtually all ASD cases were captured for downstream differential diagnosis. Feature importance analysis (Figure 2D) confirmed convergence across Gini importance, permutation-based accuracy decrease, and linear coefficients, with items P07 (social attention), P11 (communicative initiative), and P12 (social engagement) consistently ranked among the top discriminative features. Validation benchmarking (Figure 2C and E) demonstrated that the digital P-item model substantially outperformed the demographics-only baseline and approached clinical gold-standard performance, indicating the potential of the gamified digital assessment to serve (future application requiring prospective external validation) as a first-line screening aid in settings where trained clinical administration is unavailable. We caution, however, that the stage 1 ASD-ND discrimination may partly reflect broad cognitive-developmental differences rather than social cognition specifically. FSIQ values were available only for a subset of clinical participants (ASD: 28/51; mean 66.3, SD 19.1; SCD: 50/54; mean 78.7, SD 17.4) and were not measured in any ND participant (0/95). Consequently, a direct ASD-ND IQ comparison and IQ-adjusted stage 1 analysis were not possible, and no ASD-ND IQ effect size has been reported. Normative FSIQ values imputed for the ND group were used only in an exploratory supplementary check and have not been treated as measured data. The stage 1 estimates should therefore be interpreted as a composite signal reflecting both social cognitive and broader cognitive-developmental differences, pending replication in IQ-matched cohorts.

**Figure 3.**
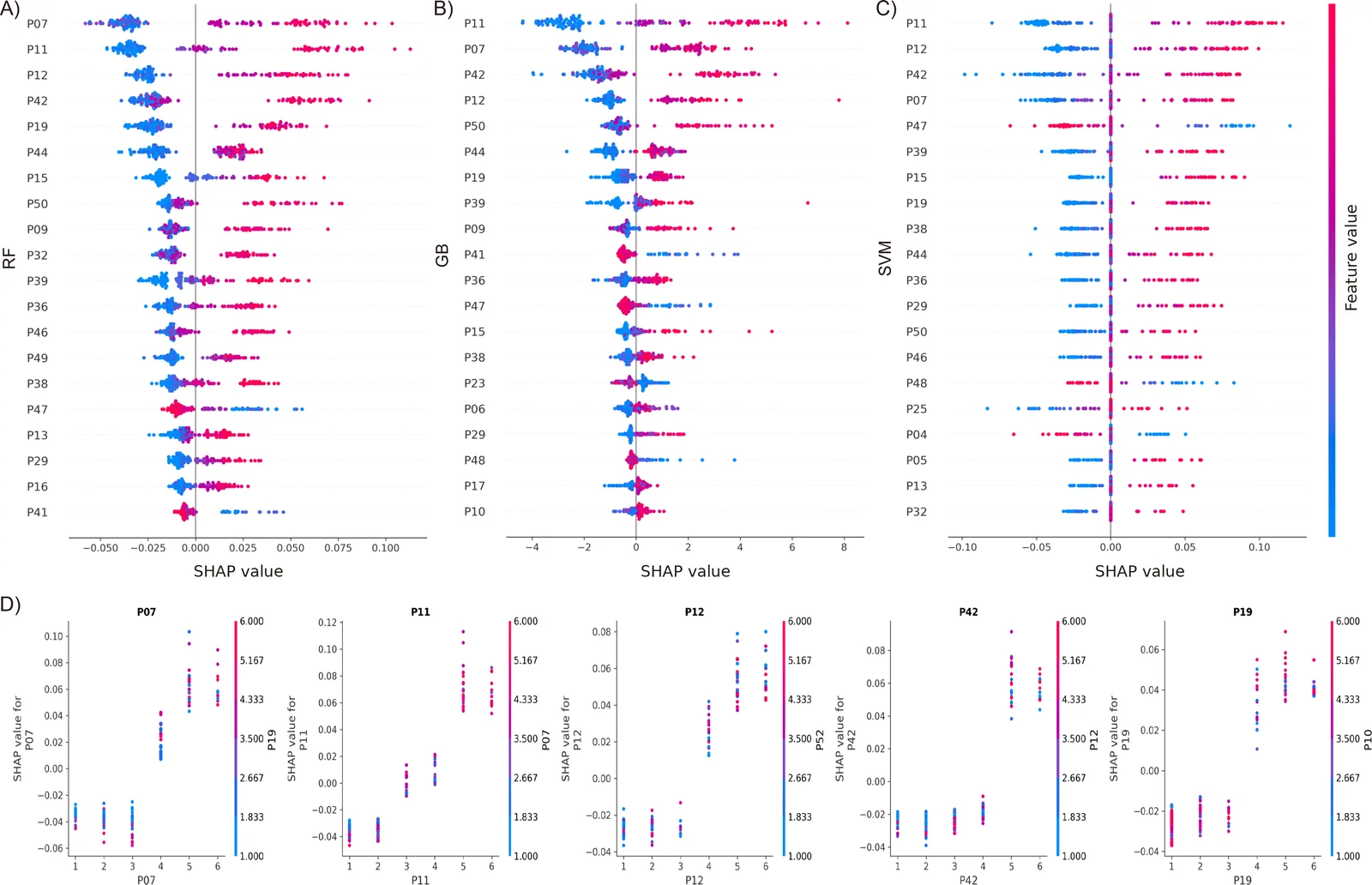
Shapley Additive Explanations (SHAP) explainability analysis for stage 1 digital biomarkers (autism spectrum disorder [ASD] versus neurotypically developing [ND]). (A-C) SHAP beeswarm plots for (A) random forest (RF), (B) gradient boosting (GB), and (C) support vector machine (SVM), displaying the top 15 buddy plan items ranked by mean absolute SHAP value. Each point represents a participant. The horizontal position indicates SHAP contribution to the ASD prediction (positive=toward ASD; negative=toward ND), and color encodes the feature value (red=high social difficulty; blue=low social difficulty). Five consensus biomarkers—P07 (social attention), P11 (communicative initiative), P12 (social engagement), P42 (empathy and theory of mind), and P19 (social motivation)—consistently ranked among the top features across all 3 algorithms, exhibiting monotonic positive directionality. (D) Individual SHAP dependence plots for each of the 5 consensus biomarkers showing the nonlinear relationship between item score and SHAP value. The vertical spread at each feature value reflects interaction effects with other features.

SHAP beeswarm plots (Figure 3A-C) revealed consistent cross-algorithm convergence among the top-ranked features. Across RF, GB, and SVM, items P07 (social attention), P11 (communicative initiative), P12 (social engagement), P42 (empathy and ToM), and P19 (social motivation) emerged as consensus biomarkers, each ranking within the top 10 features in at least 3 of the 4 algorithms (Table S3 in Multimedia Appendix 2). All 5 biomarkers exhibited monotonic positive SHAP directionality: higher social difficulty scores (red points) were associated with positive SHAP values, pushing predictions toward the ASD class, while lower difficulty scores (blue points) pushed predictions toward the ND class. This monotonic relationship was confirmed by the individual SHAP dependence plots (Figure 3D), which further revealed nonlinear dose-response patterns for certain biomarkers. To formally validate the discriminative contribution of consensus biomarkers, Mann-Whitney *U* tests were performed comparing SHAP value distributions between the ASD and ND groups for each of the 5 consensus biomarkers. All 5 biomarkers showed statistically significant between-group SHAP differences (P07: *U*=3842; *P*<.001; P11: *U*=3651; *P*<.001; P12: *U*=3498; *P*<.001; P42: *U*=3215; *P*<.001; P19: *U*=2987; *P*<.001; all Bonferroni-corrected *P*<.01), confirming group-level differences in feature contributions.

### Stage 2: Buddy Drill Differential Diagnosis

In the originally reported analysis, in which correlation-based item selection had been performed on the full ASD-SCD sample before cross-validation, LR appeared to achieve a nested AUC of 0.767. This estimate was, however, inflated by feature-selection leakage. When item ranking, selection, and imputation were repeated entirely within each outer training fold (a fully nested pipeline), discrimination fell to chance across all algorithms: LR nested AUC of 0.62 (95% CI 0.51‐0.74; sensitivity 0.61; specificity 0.71), SVM AUC of 0.61, RF AUC of 0.60, and GB AUC of 0.52, each with a 95% CI whose lower bound was at or near 0.50. Stage 2 therefore did not robustly differentiate ASD from SCD in this sample.

As a clinician-rated benchmark, a multivariable model of the observer scales did differentiate the 2 conditions: combining all SRS-2 and VABS subscales achieved a leakage-free nested AUC of 0.77 (95% CI 0.68‐0.86), and the SRS-2 restricted/repetitive-behavior subscale showed the largest single group difference (ASD vs SCD Cohen *d*=0.84). By contrast, the corresponding digital restricted/repetitive-behavior proxy (Buddy Plan F4 items) did not differentiate the groups (nested AUC 0.55, 95% CI 0.44‐0.66). Because the clinician-rated scales are not independent of the diagnostic process and require trained administration, they are reported only as a benchmark and not as a stand-alone or digital differentiator.

**Figure 4.**
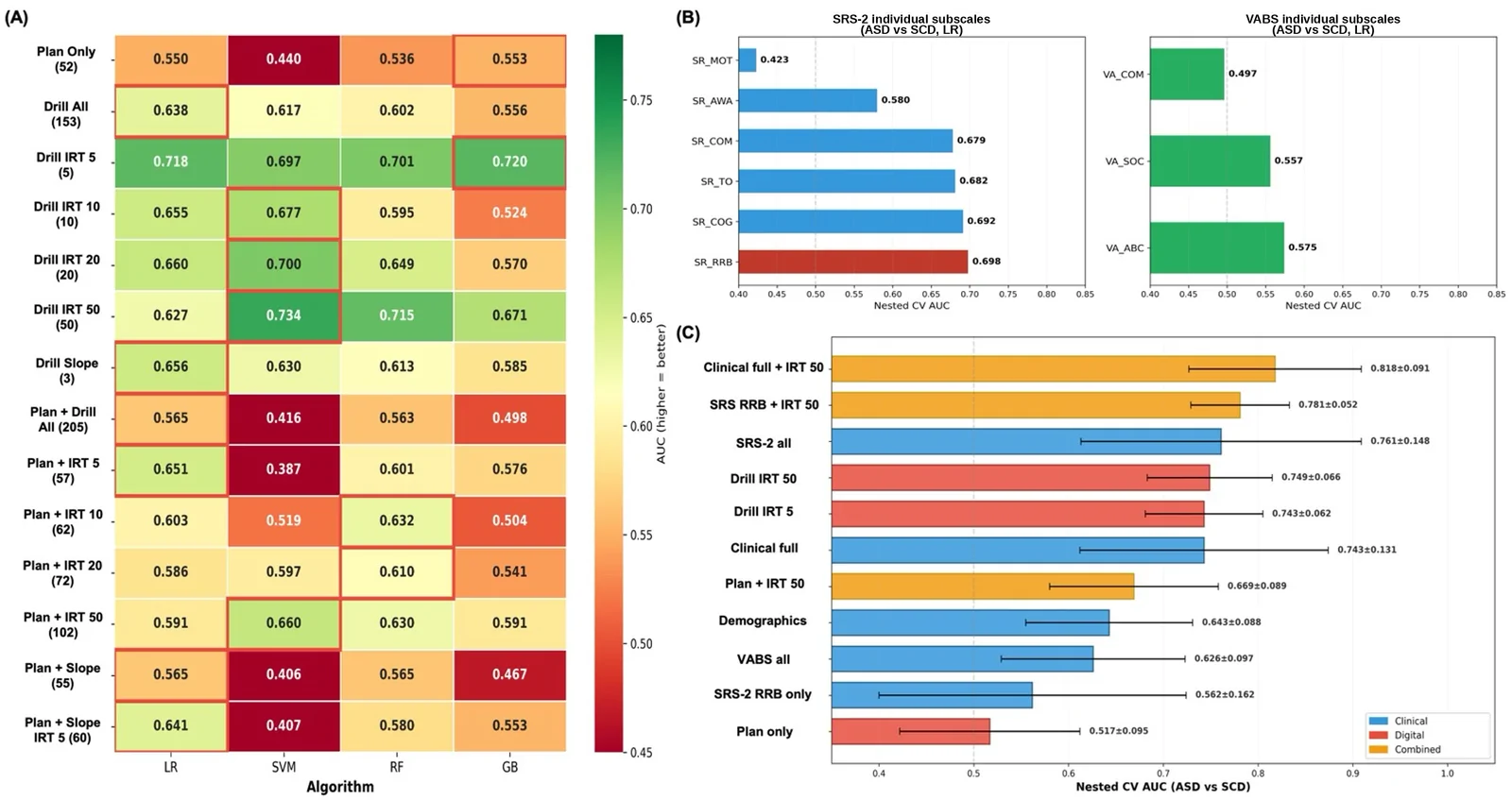
Stage 2 differential diagnosis performance and feature incompatibility analysis (autism spectrum disorder [ASD] versus social communication disorder [SCD]). The analysis comprised 100 participants (ASD: 51; SCD: 49). (A) Heatmap of nested cross-validation (CV) area under the receiver operating characteristic curve (AUC) values across 4 algorithms (logistic regression [LR], random forest [RF], gradient boosting [GB], and support vector machine [SVM]) and multiple feature set configurations, including Buddy Plan items only (P), psychometrically selected Buddy Drill items (D:IRT), full Buddy Drill items (D:All), and combined feature sets (P+D). Color gradient ranges from red (low AUC) to green (high AUC). (B) Comparative bar plots of individual Social Responsiveness Scale-2 (SRS-2) and Vineland Adaptive Behavior Scales (VABS) subscale AUC values for ASD versus SCD classification. (C) Horizontal bar chart comparing nested AUC values across feature set configurations for the best-performing algorithm. These panels reflect the originally reported analysis with full sample feature selection and are superseded by the fully nested, leakage-free estimates reported in the text (all AUC≈0.5‐0.6). IRT: item response theory.

Under the fully nested pipeline, no feature configuration exceeded chance for ASD-SCD differentiation (Figure 4A): self-report P-items alone yielded a nested AUC of 0.55 (95% CI 0.44‐0.67), objective D-items yielded an AUC of 0.62 (95% CI 0.51‐0.74), and the combined P+D set yielded an AUC of 0.63 (95% CI 0.51‐0.74). The combined set performed no worse than D-items alone. The “information dilution” effect reported previously—in which adding P-items appeared to degrade performance—therefore did not persist once selection leakage was removed and is now attributed to that leakage. Consistent with these results, no single SRS-2 or VABS subscale discriminated ASD from SCD (all AUC <0.65; Figure 4B), underscoring the genuine difficulty of this diagnostic boundary. An abbreviated 5-item variant, evaluated within the same fully nested framework, likewise remained at chance for ASD-SCD differentiation (nested AUC 0.60, 95% CI 0.49‐0.70; Table 2) and is therefore not recommended as a rapid-screening tool for this purpose.

**Table 2.** Comparison of IRT^a^-50 and IRT-5 protocols^b^ for stage 2 ASD^c^ versus SCD^d^ differential diagnosis (N=100; ASD: n=51, SCD: n=49).

Metric	IRT-50 (50 items)^e^	IRT-5 (5 items)^e^	Δ (IRT-5−IRT-50)
Nested AUC^f^ (95% CI)	0.62 (0.51-0.74)	0.60 (0.49-0.70)	−0.02
Sensitivity^g^, % (n/N)	61 (31/51)	57 (29/51)	−4 pp^h^
Specificity^i^, % (n/N)	71 (35/49)	55 (27/49)	−16 pp
Concordance with IRT-50, % (n/N)	—^j^	85 (85/100)	—
Administration time^k^	Approximately 15‐20 min	Approximately 2‐3 min	Approximately 85% reduction

a IRT: item response theory.

b Both protocols performed at or near chance level for ASD versus SCD differentiation; the table is retained to document the abbreviated protocol comparison and not to support differential diagnostic use.

c ASD: autism spectrum disorder.

d SCD: social communication disorder.

e All values are from a single, fully nested, leakage-free 5×5 cross-validation pipeline in which item ranking, selection, and median imputation were performed within each outer training fold; the classifier was regularized logistic regression. Operating characteristics are reported at a .50 probability threshold.

f AUC: area under the receiver operating characteristic curve.

g Sensitivity denominator=51 (ASD).

h pp: percentage points.

i Specificity denominator=49 (SCD).

j Not applicable.

k Administration time estimates assume an average pace of 20-25 s per item.

## Discussion

### Principal Findings

Relative to the aims stated at the end of the Introduction, this study produced a clear dissociation between the 2 stages of the gamified pipeline. The Buddy Plan screening module distinguished children with ASD from their ND peers with high internal accuracy, supporting its promise as a screening aid. This result must nonetheless be interpreted cautiously because the 2 groups differed substantially in general cognitive ability that could not be equated in the present design. The Buddy Drill differentiation module, in contrast, did not reliably separate ASD from SCD once a fully nested analysis pipeline was applied, and the objective task performance, self-report rating, or their combination did not perform above chance. An apparent advantage of objective over self-report features and a related information-dilution pattern that had appeared in an earlier, nonnested analysis did not survive leakage-free reanalysis and are therefore not interpreted as genuine effects. Screening performance was stable across developmental age subgroups, and the explainability analysis highlighted candidate, model-derived features that require independent confirmation.

These findings help localize where the signal that distinguishes ASD from SCD resides. The 2 conditions are separated in DSM-5 primarily by RRBs [3], and, consistent with this, a clinician-rated restricted/repetitive-behavior scale showed a substantial group difference. Moreover, a multivariable clinician-rated model differentiated the conditions, whereas the gamified digital tasks did not: the objective social-judgment scenarios, self-report items, or digital restricted/repetitive-behavior proxy did not exceed chance. This dissociation suggests that the present gamified tasks index the social-communication difficulty shared by ASD and SCD rather than the restricted/repetitive-behavior dimension that separates them. A central design implication is that a digital differential diagnostic tool will likely need to measure RRBs and sensory features directly and sensitively—for example, through dedicated interactive tasks or caregiver-facing digital modules—rather than relying on social-judgment performance alone. The clinician-rated benchmark must be interpreted cautiously, because those measures are not independent of the diagnostic decision and require trained raters; it is presented to explain the digital null result, not as evidence that the present instrument differentiates the conditions.

For the screening stage, the decision threshold was set to favor sensitivity over specificity, a tradeoff that is appropriate for first-line screening, in which a missed case carries greater downstream cost than a false-positive referral that can be resolved by subsequent assessment [1,14]. Under this configuration, the self-report module approached the accuracy of a clinician-administered reference standard while requiring no clinician involvement, a property that is potentially attractive for resource-limited or nonspecialist settings [8,9]. This comparison should nonetheless be regarded as preliminary given the cognitive-ability imbalance between the groups. Because screening performance was also stable across developmental age subgroups, the module appears reasonably robust to maturational variation within the age range studied, although replication in independent, IQ-matched cohorts remains necessary before any screening use can be recommended.

The explainability analysis supported the face validity of the screening model without establishing a mechanism. A small set of items recurred as the most influential features across several algorithmically distinct classifiers, and the direction of their effects aligned with clinical expectation, including nonlinear patterns for an empathy and ToM item that are consistent with compensatory processes described in the autism literature [25,26]. Because SHAP can quantify each feature’s contribution to model predictions but cannot demonstrate causal or mechanistic roles [22], these converging items are best regarded as candidate, model-derived markers that require independent, prospective confirmation rather than as validated clinical mechanisms.

The difficulty of the differentiation stage is consistent with the clinical and nosological overlap between the 2 conditions. ASD and SCD share the core domain of social-communication impairment and are separated in DSM-5 chiefly by the presence of RRBs [3], a domain that neither gamified module was designed to measure. Self-report ratings and social-judgment tasks that probe the shared social-communication phenotype may therefore index the common pathway [27] through which both conditions manifest, rather than the features that distinguish them. Consistent with this account, the between-group difference on directly observed interaction behavior in our sample was negligible, and observer-rated social measures alone are known to have limited power to separate these overlapping presentations [7]. We note that an earlier analysis had interpreted differences among feature sets as evidence of a construct-level information-dilution effect. Because that pattern did not survive leakage-free reanalysis, we no longer advance it and instead attribute the earlier impression to analytic bias rather than to a genuine property of the constructs.

Across all 4 algorithm classes, no feature configuration robustly separated ASD from SCD once selection leakage was removed, and individual clinician-rated subscales likewise failed to reach a useful level of accuracy for discriminating the 2 conditions. Together, these results indicate that the ASD-SCD boundary—defined in DSM-5 primarily by RRBs, which neither module directly measures—could not be resolved by the present gamified tasks in a sample of this size. The earlier impression that psychometric item selection was central to differential performance reflected optimistic bias introduced when items were selected on the full dataset rather than within cross-validation folds, and not a generalizable signal. Future work will require substantially larger, IQ- and language-matched samples; direct measurement of RRBs; and preregistered, fully nested analysis pipelines before differential diagnostic claims can be supported.

Methodologically, the contrast between our earlier and reanalyzed results is an instructive cautionary example for the digital-biomarker field. When correlation-based item selection and imputation were carried out on the full sample before cross-validation, the differentiation model appeared informative. When the identical steps were nested within each training fold, the apparent advantage of objective features over self-report features disappeared, and all configurations reverted to chance. This is a well-characterized consequence of performing supervised feature selection outside the resampling loop, which permits information from held-out cases to influence model construction and inflates apparent performance, especially in small samples [21,28]. The episode underscores that feature selection, imputation, and any data-driven dimensionality reduction must be embedded within cross-validation and that preregistration of the analysis plan provides useful protection against such optimistic bias.

Buddy Drill was designed to probe social-cognitive processes, motivated by accounts in which ASD involves impaired mentalizing while SCD shows relatively preserved mentalizing with selective pragmatic deficits [11,12,29]. We had noted item-level accuracy differences on individual scenarios (eg, D099 and D075). However, because these item-level features were identified using the full sample rather than within cross-validation folds, they are considered exploratory and were not confirmed in the leakage-free analysis. We therefore have not interpreted them as validated differential biomarkers, and the ToM framing of Buddy Drill remains a hypothesis rather than a supported finding in these data.

From a clinical standpoint, these results temper expectations for gamified social-cognitive tasks as stand-alone differential diagnostic tools. Because the information that distinguishes ASD from SCD appears to lie in RRBs as characterized by trained clinicians [3], and because such ratings are neither independent of the diagnostic decision nor scalable without professional administration, they cannot substitute for an automated digital instrument. The practical corollary is that a useful digital differentiation tool would need to elicit RRBs and sensory features directly and sensitively, and would then require prospective, externally validated evaluation before any clinical role could be considered [30].

The variability of some algorithms across resampling folds further illustrates how fragile differential estimates can be at the present sample size, where a single classifier may appear either strong or uninformative depending on how the folds are partitioned. Such instability reinforces the importance of reporting nested, resampled estimates with CIs rather than single point values and the importance of interpreting abbreviated or reduced-item protocols with corresponding caution [21]. In our data, shortened item sets conferred no advantage for differentiation once leakage was removed, and we therefore do not recommend them for that purpose in the absence of substantially larger validation samples.

### Comparison With Prior Work

Previous digital screening studies have primarily focused on ASD versus typically developing comparisons, reporting AUC values of 0.85‐0.95 [14-16]. Our stage 1 result (AUC=0.912) is consistent with this range while adding nested cross-validation and SHAP explainability. The ASD-SCD differential comparison addressed in stage 2 has received less attention in the digital biomarker literature. To our knowledge, this is the first gamified assessment system to target this specific differential. We initially observed what appeared to be an information-dilution effect, but this did not survive a fully nested reanalysis and is now attributed to feature-selection leakage. We therefore do not advance it as a substantive finding. The more robust implication is methodological: in small digital-phenotyping samples, feature selection and dimensionality reduction must be nested within cross-validation, as emphasized in the ML literature [28], or estimates will be optimistically biased.

Our secondary analyses drew on a dimensionality-reduction framework previously applied to gamified emotional-cognitive indices in children [23], from which we derived composite “N-score” dimensions for the screening comparison and an exploratory hybrid model for differentiation. Because these composites and their accompanying item selection were derived on the full sample rather than within cross-validation folds, they are subject to the same optimistic bias identified for the primary differentiation analysis. We therefore report them only as hypothesis-generating and do not interpret them as validated evidence of differential diagnostic value. The dimensionality-reduction approach may nonetheless merit re-examination in adequately powered future studies that embed every data-driven step within the resampling procedure.

### Limitations

This study has some limitations. First, several sample and measurement constraints limit interpretation. The Buddy Drill module was not administered to the ND group, and thus, stage 2 could be evaluated only for the ASD-versus-SCD contrast. Moreover, whether the objective items discriminate children with ASD from typically developing children remains untested. More importantly, cognitive ability was not comparable across groups and could not be adjusted for: FSIQ was measured only in subsets of the clinical groups and not in the neurotypical group, and thus, no measured ASD-ND IQ comparison or IQ-matched stage 1 analysis was possible. Stage 1 accuracy may partly reflect general cognitive-developmental differences rather than social cognition specifically, and language ability was likewise not assessed. In addition, Buddy Plan involves self-report, which raises validity concerns for younger and lower-ability children, and no test-retest reliability or measurement-invariance data were collected.

Second, analytic and generalizability constraints apply. The originally reported stage 2 estimate was inflated by feature-selection leakage. When item selection and imputation were nested within each training fold, discrimination fell to chance, and we therefore have reported stage 2 as a null result and have treated all secondary analyses that relied on full-sample selection or PCA (N-scores and hybrid models) as exploratory. The item-selection procedure was also only a correlation-based approximation of IRT rather than formal IRT modeling. All results were derived from internal cross-validation in a single, predominantly male, culturally homogeneous Korean sample without external validation, which limits generalizability. Moreover, because stage 2 did not exceed chance, it has no established clinical utility for ASD-SCD differentiation, and thus, predictive values based on the earlier (leaky) estimates are not reported.

Third, some interpretive and governance caveats remain. The Buddy Plan subscale structure was derived by expert consensus without factor-analytic validation, and one subscale showed low internal consistency. Thus, subscale-level interpretations are provisional. Likewise, the SHAP-based explainability analyses are associational rather than causal, and the highlighted features should be regarded as candidate markers requiring independent validation. Finally, 2 authors are affiliated with the developer of the assessment platform. Although the analyses were conducted independently at the Korea Brain Research Institute under a prespecified plan, no independent data-monitoring committee was involved, and the study was not preregistered. These factors warrant consideration when interpreting the findings.

### Conclusions

The gamified Buddy Plan screening module distinguished children with ASD from their ND peers with high internal accuracy, supporting its promise as a first-line screening aid. However, this result is confounded by an unmatched cognitive-ability difference between the groups and therefore remains preliminary. The Buddy Drill differentiation module, by contrast, did not reliably distinguish ASD from SCD once feature-selection leakage was removed with a fully nested pipeline. Beyond the specific instrument, the study carries a broader message for the digital-biomarker field: in small clinical samples, feature selection and dimensionality reduction must be nested within cross-validation because procedures applied outside it can create apparent differential signals that do not generalize. Clinically, the observation that the differential signal between ASD and SCD resided in clinician-rated RRBs rather than in social-judgment task performance suggests that future digital differentiation tools should measure RRBs and sensory features directly. Prospective, externally validated studies with IQ- and language-matched, sex-balanced cohorts will be required before any clinical application, and no clinical deployment is warranted on the basis of the present data.

## Supplementary material

10.2196/102714 Multimedia Appendix 1 Participant flow diagram (Journal Article Reporting Standards [JARS] format).

10.2196/102714 Multimedia Appendix 2 Buddy Plan subscale structure, internal consistency, and Shapley Additive Explanations–subscale convergence.

10.2196/102714 Multimedia Appendix 3 Mathematical formulation of the N-score derivation pipeline.

## Abbreviations

ADHD: attention-deficit/hyperactivity disorder
ASD: autism spectrum disorder
AUC: area under the receiver operating characteristic curve
CREMLS: Consolidated Reporting Guidelines for Machine Learning Modeling Studies
DIF: differential item functioning
DSM-5: Diagnostic and Statistical Manual of Mental Disorders, Fifth Edition
FSIQ: full-scale IQ
GB: gradient boosting
HR: high risk
IRB: Institutional Review Board
IRT: item response theory
JARS: Journal Article Reporting Standards
LR: logistic regression
MCAR: missing completely at random
ML: machine learning
N-scores: neurodevelopmental scores
ND: neurotypically developing
PCA: principal component analysis
RF: random forest
RRB: restricted and repetitive behavior
SCD: social communication disorder
SHAP: Shapley Additive Explanations
SRS-2: Social Responsiveness Scale-2
SVM: support vector machine
ToM: theory of mind
VABS: Vineland Adaptive Behavior Scales
