# Supplementary material for: Two-Stage Gamified Digital Assessment for Autism Spectrum Disorder Screening and the Limits of Differentiating Social Communication Disorder in Children and Adolescents: Cross-Sectional Diagnostic Accuracy Study Using Explainable Machine Learning
Source: JMIR Serious Games. 2026 Sep 23;14:e102714. doi: 10.2196/102714 (PMC13600596; doi:10.2196/102714)
Supplement: Supplementary file 1 [file games-v14-e102714-s001.docx]

**Multimedia Appendix 1.** Participant flow diagram (Journal Article Reporting Standards [JARS] format). Cross-sectional diagnostic accuracy study of a two-stage gamified digital assessment for autism spectrum disorder (ASD) screening and differentiation from social communication disorder (SCD) in 275 children and adolescents aged 6–18 years recruited at 5 clinical and community sites in the Republic of Korea (May 2024–February 2025). All enrolled participants completed the Stage 1 module (Buddy Plan; 52 self-report P-items); the Stage 1 screening analysis (ASD vs ND; n=146) used nested 5×5 cross-validation with a random forest primary model. The Stage 2 module (Buddy Drill; 153 story-based D-items) was administered to the clinical and high-risk groups only (n=180); the ND group was not administered (missing by design). The Stage 2 differential-diagnosis analysis (ASD vs SCD; n=100; ASD 51, SCD 49) used regularized logistic regression on 50 item response theory (IRT)-selected items; in a fully nested, leakage-free re-analysis, Stage 2 discrimination did not exceed chance (nested AUC ≈ 0.62; 95% CI including 0.50). Groups shown to the right of each stage were not part of the primary classification analyses and were used only in cross-group generalization analyses. ADHD: attention-deficit/hyperactivity disorder; AUC: area under the receiver operating characteristic curve; CI: confidence interval; CV: cross-validation; DSM-5: Diagnostic and Statistical Manual of Mental Disorders, Fifth Edition; GB: gradient boosting; HR: high risk; IRT: item response theory; LR: logistic regression; ND: neurotypically developing; PCA: principal component analysis; RF: random forest; SHAP: SHapley Additive exPlanations; SVM: support vector machine.
