# Supplementary material for: Two-Stage Gamified Digital Assessment for Autism Spectrum Disorder Screening and the Limits of Differentiating Social Communication Disorder in Children and Adolescents: Cross-Sectional Diagnostic Accuracy Study Using Explainable Machine Learning
Source: JMIR Serious Games. 2026 Sep 23;14:e102714. doi: 10.2196/102714 (PMC13600596; doi:10.2196/102714)
Supplement: Supplementary file 2 [file games-v14-e102714-s002.docx]

**Multimedia Appendix 2.** Buddy plan subscale structure, internal consistency, and Shapley Additive Explanations–subscale convergence

**A. Five-factor subscale framework**

The 52 Buddy Plan items were organized into 5 theoretically derived subscales based on expert consensus classification by a panel of board-certified child psychiatrists and developmental psychologists, drawing on established frameworks from the social cognition, social communication, and neurodevelopmental literature. The resulting 5-factor structure captures complementary dimensions of social-communicative functioning:

(1) Factor 1 (F1) — Social Cognition and Contextual Understanding (19 items): items assessing theory of mind reasoning, inference of others’ intentions, comprehension of figurative and idiomatic language, and social awareness.

(2) Factor 2 (F2) — Interaction Skills and Nonverbal Communication (11 items): items evaluating eye contact maintenance, proxemics, vocal prosody modulation, and the ability to read and respond to affective signals.

(3) Factor 3 (F3) — Social Motivation and Avoidance/Anxiety (9 items): items capturing approach–avoidance tendencies toward peer and group interactions, social anxiety, and preference for solitary activities.

(4) Factor 4 (F4) — Repetitive/Restricted Interests and Sensory Rigidity (6 items): items measuring perseverative interests, stereotyped motor behaviors, sensory sensitivities, and intolerance of change.

(5) Factor 5 (F5) — Self-Expression and Self-Regulation (7 items): items assessing response latency, emotional expression, self-awareness of strengths and weaknesses, and symbolic play capacity.

This classification is theoretically motivated rather than empirically derived; no exploratory factor analysis (EFA) or confirmatory factor analysis (CFA) was performed to validate the 5-factor structure statistically. Accordingly, the subscale grouping should be interpreted as a clinically informed organizational framework, and the factor solution may differ from data-driven approaches. Internal consistency was assessed using Cronbach α and McDonald ω total (Table S1).

**B. SHAP-subscale convergence and clinical interpretability**

Mapping the 5 cross-algorithm consensus SHAP biomarkers (identified in the main analysis) to the Buddy Plan subscale structure revealed a striking pattern of convergence. Three of the 5 top-ranked biomarkers belonged to F1 (Social Cognition and Contextual Understanding) — P07 (social attention), P12 (social engagement), and P42 (empathy and theory of mind) — and the remaining 2 belonged to F4 (Repetitive/Restricted Interests and Sensory Rigidity) — P11 (communicative initiative) and P19 (social motivation). No consensus biomarker originated from F2 (Interaction Skills), F3 (Social Motivation/Anxiety), or F5 (Self-Expression). Buddy Plan subscale scores by diagnostic group are presented in Table 1 of the main manuscript, which integrates demographic, clinical, and subscale-level characteristics.

For Stage 1 (ASD vs ND), F4 (Repetitive/Restricted Interests) exhibited the largest group difference (Δ = +1.391), followed by F1 (Social Cognition; Δ = +1.250). The 2 subscales contributing the largest mean differences were the same 2 subscales from which all 5 SHAP consensus biomarkers originated, providing convergent evidence that the machine-learning algorithm independently prioritized the most clinically discriminative constructs. F2 (Interaction Skills) showed a moderate difference (Δ = +0.671) but contributed no consensus biomarkers, suggesting that nonverbal interaction deficits, while present, are less diagnostically specific than social-cognitive and RRB-related features.

For the ASD–SCD comparison (Stage 2), a particularly informative pattern emerged. F2 (Interaction Skills) showed virtually no difference between ASD and SCD (Δ = −0.007), and F5 (Self-Expression) was similarly indistinguishable (Δ = −0.013). These near-zero differences confirm that ASD and SCD present identically on observable interaction behaviors — the very constructs measured by conventional clinical instruments such as the SRS-2 — providing a subscale-level explanation for the near-chance AUC (0.553) of P-items alone in Stage 2 differential diagnosis. By contrast, F4 (RRB/Sensory) showed the largest ASD–SCD difference (Δ = +0.266), consistent with the DSM-5 criterion that restricted and repetitive behaviors primarily distinguish ASD from SCD [3 in main manuscript]. Notably, the HR group exhibited the highest F3 (Social Motivation/Anxiety) scores (mean 3.577), exceeding even the ASD group (3.453), suggesting that social avoidance and anxiety in HR participants may reflect a qualitatively distinct mechanism from the social-cognitive deficits characteristic of ASD.

**Table S1. Buddy Plan subscale structure, item distribution, and internal consistency.**

| **Factor** | **Subscale Label** | **Items (n)** | **Representative Items** | ***α*** | ***ω*** |
| --- | --- | --- | --- | --- | --- |
| **F1** | Social Cognition & Contextual Understanding | 19 | P07, P12, P32, P42, P49 | .871 | .892 |
| **F2** | Interaction Skills & Nonverbal Communication | 11 | P30, P37, P43, P45, P47 | .806 | .866 |
| **F3** | Social Motivation & Avoidance/Anxiety | 9 | P01, P10, P18, P29, P34 | .666 | .761 |
| **F4** | Repetitive/Restricted Interests & Sensory Rigidity | 6 | P04, P11, P13, P19, P22 | .688 | .794 |
| **F5** | Self-Expression & Self-Regulation | 7 | P03, P05, P25, P26, P35 | .508 | .689 |
| **Total** | *Buddy Plan composite (all items)* | **52** | — | — | — |

α = Cronbach alpha; ω = McDonald omega total. F1 and F2 demonstrated good internal consistency (α > .80). F3 and F4 demonstrated acceptable reliability (α > .65; ω > .76). F5 showed lower α (.508), below the conventional acceptability threshold of α = .70. The improved ω (.689) suggests moderate composite reliability when accounting for non-tau-equivalent item loadings; F5 results should therefore be interpreted with caution. A post hoc sensitivity analysis excluding all 7 F5 items from the Stage 1 feature set yielded negligible performance change (RF AUC = 0.905 vs 0.912 with full items), indicating that F5 items contribute minimally to screening accuracy and could be removed without substantive diagnostic loss. The F5 subscale label and item grouping should be revisited in future EFA/CFA studies.

**Table S2. Stage 1 nested cross-validation results across four machine-learning algorithms (N = 146; ASD = 51, ND = 95).**

| **Metric** | **RF** | **SVM (linear)** | **GB** | **LR** |
| --- | --- | --- | --- | --- |
| **Nested AUC** | **0.912** | 0.885 | 0.869 | 0.826 |
| SD | 0.054 | 0.097 | 0.066 | 0.093 |
| PR-AUC | 0.869 | 0.802 | 0.836 | 0.710 |
| F1 score | 0.744 | 0.716 | 0.739 | 0.703 |
| Sensitivity | 0.627 | 0.667 | 0.667 | 0.627 |
| Specificity | 0.968 | 0.895 | 0.926 | 0.916 |
| Best parameters (mode) | n_est=300; max_features=log2; min_leaf=2 | C=0.01; kernel=linear | lr=0.2; depth=5; subsample=0.8 | C=0.01; L2 |

All values reflect outer-fold predictions that were never used for hyperparameter tuning. Hyperparameters were selected by inner-fold GridSearchCV; the mode across 5 outer folds is reported. AUC = area under the receiver operating characteristic curve; GB = gradient boosting; LR = logistic regression; PR-AUC = area under the precision-recall curve; RF = random forest; SVM = support vector machine.

**Table S3. Cross-stage digital biomarker convergence analysis.**

| **Stage** | **Item** | **RF SHAP** | **GB SHAP** | **SVM SHAP** | **IRT Rank** | **Construct** | **Key Finding** |
| --- | --- | --- | --- | --- | --- | --- | --- |
| **Stage 1 (ASD vs ND)** | **P07** | #1 | #2 | #4 | — | Social attention | Sensitivity to nonverbal cues |
|  | **P11** | #2 | #1 | #1 | — | Communicative initiative | Ability to initiate social interactions |
|  | **P12** | #3 | #4 | #2 | — | Social engagement | Active participation in social exchanges |
|  | **P42** | #4 | #3 | #3 | — | Empathy / ToM | Cognitive empathy; inferring others’ mental states |
|  | **P19** | #5 | #5 | #8 | — | Social motivation | Drive to establish relationships |
| **Stage 2 (ASD vs SCD)** | **D099** | #3 | — | #3 | #1 | Δ accuracy: −22.7% | SCD 95.6% vs ASD 72.9%; strongest triple-convergent biomarker (★★★) |
|  | **D025** | #4 | — | #10 | #7 | Δ accuracy: −16.4% | Triple convergence across IRT, RF, SVM (★★★) |
|  | **D075** | #1 | — | — | #6 | Δ accuracy: +20.1% | Reversed: ASD > SCD; literalistic-processing advantage (★★) |
|  | **D146** | #5 | — | #4 | — | — | Dual RF+SVM convergence (★★) |
|  | **D112** | #8 | — | #14 | #5 | Δ accuracy: −21.0% | IRT–RF convergence (★★) |

Stage 1: rankings reflect mean |SHAP| across all samples; P07, P11, P12, and P42 ranked in the top 4 across all 3 algorithms. Stage 2: IRT rank = item response theory discrimination parameter ranking (approximated via point-biserial correlations); Δ accuracy = SCD accuracy − ASD accuracy (negative = SCD outperforms ASD). Convergence rating: ★★★ = triple convergence (IRT + RF + SVM); ★★ = dual convergence. D075 uniquely shows ASD > SCD (+20.1%), suggesting a literalistic-processing advantage consistent with the weak central coherence account. GB = gradient boosting; IRT = item response theory; RF = random forest; SHAP = SHapley Additive exPlanations; SVM = support vector machine; ToM = theory of mind.

**Table S4. Stage 2 nested cross-validation: ASD versus SCD using 50 IRT-selected Buddy Drill items (N = 104; ASD = 51, SCD = 53).**

| **Metric** | **LR (L2)** | **RF** | **GB** | **SVM (RBF)** |
| --- | --- | --- | --- | --- |
| **Nested AUC** | **0.767** | 0.727 | 0.701 | 0.551 |
| SD | 0.111 | 0.099 | 0.115 | 0.315 |
| PR-AUC | 0.779 | 0.708 | 0.708 | 0.615 |
| F1 score | 0.727 | 0.694 | 0.617 | 0.661 |
| Sensitivity | 0.706 | 0.667 | 0.569 | 0.824 |
| Specificity | 0.755 | 0.735 | 0.714 | 0.306 |
| Best parameters (mode) | C=0.001; L2 | n_est=200; max_features=sqrt | lr=0.1; depth=3 | C=1; γ=scale; RBF |

LR achieves the highest AUC (0.767) with the lowest variance (SD = 0.111). SVM (RBF kernel) shows extreme instability (SD = 0.315; fold AUCs ranged 0.20–1.00), illustrating the importance of nested cross-validation for unbiased performance estimation in small clinical samples. AUC = area under the receiver operating characteristic curve; GB = gradient boosting; IRT = item response theory (approximated via point-biserial correlations); LR = logistic regression; PR-AUC = area under the precision-recall curve; RBF = radial basis function; RF = random forest; SVM = support vector machine.

**Table S5. Clinical use case summary across pipeline configurations.**

|  | **Stage 1 Screening** | **Stage 2 Differential** | **Rapid Protocol (IRT-5)** |
| --- | --- | --- | --- |
| **Configuration** | Buddy Plan 52 P-items; RF; threshold = 0.200 | Buddy Drill IRT-50; LR (C = 0.001, L2) | Buddy Drill IRT-5; LR |
| **Nested AUC** | **0.912** | **0.767** | **0.720** |
| **Rationale** | 96.1% sensitivity; only 2/51 ASD missed; high NPV at modest prevalence | Best AUC with lowest variance; interpretable linear model | Administration ~2–3 min; 85% concordance with IRT-50 |

ASD = autism spectrum disorder; AUC = area under the receiver operating characteristic curve; IRT = item response theory; LR = logistic regression; ND = neurotypically developing; NPV = negative predictive value; RF = random forest. The IRT-5 abbreviated protocol provides a rapid-screening variant when administration time is constrained, with the trade-off of a modest reduction in sensitivity relative to IRT-50.
