# Supplementary material for: Two-Stage Gamified Digital Assessment for Autism Spectrum Disorder Screening and the Limits of Differentiating Social Communication Disorder in Children and Adolescents: Cross-Sectional Diagnostic Accuracy Study Using Explainable Machine Learning
Source: JMIR Serious Games. 2026 Sep 23;14:e102714. doi: 10.2196/102714 (PMC13600596; doi:10.2196/102714)
Supplement: Supplementary file 3 [file games-v14-e102714-s003.docx]

**Multimedia Appendix 3.** Mathematical formulation of the N-score derivation pipeline

This appendix presents the formal mathematical specification of the five-step pipeline used to derive composite neurodevelopmental scores (N-scores) from the 52 Buddy Plan items. Let *x_ij_* denote the raw 6-point Likert response of participant *j* (*j* = 1, …, *N*) on item *i* (*i* = 1, …, 52).

**Step 1. Item-level z-score standardization.** Each raw response is centered and scaled by its item-specific mean and standard deviation:

*z_ij_* = (*x_ij_* − μ_i_) / σ_i_	(1)

where μ_i_ and σ_i_ are the sample mean and sample standard deviation of item *i* across all *N* participants.

**Step 2. Outlier Winsorization.** To prevent extreme responses from dominating the eigendecomposition, z-scores are capped at ±2.5 SD:

z̃*_ij_* = max(−2.5, min(2.5, *z_ij_*))	(2)

**Step 3. Global PCA on the item correlation matrix.** Let **Z̃** ∈ ℝ^N×52^ denote the capped z-score data matrix. The 52×52 item correlation matrix **R** is decomposed via eigendecomposition:

**R** = (1/(*N*−1)) **Z̃**^T^ **Z̃** = **V Λ V**^T^	(3)

where **V** = [**v**_1_, …, **v**_52_] contains the orthonormal eigenvectors (item loadings), and Λ = diag(λ_1_, …, λ_52_) is the diagonal matrix of eigenvalues in descending order. Each item is projected into a 2-dimensional loading space using its coordinates (v_1_[i], v_2_[i]).

**Step 4. K-means clustering of items.** Items are partitioned into K = 3 clusters (selected via the elbow method) by minimizing the within-cluster sum of squared Euclidean distances in the 2D loading space:

{*C*_1_, *C*_2_, *C*_3_} = arg min ∑_k=1_^3^ ∑_i ∈ Ck_ ∥(v_1_[i], v_2_[i]) − μ_k_∥²	(4)

where μ_k_ denotes the 2-dimensional centroid of cluster *C*_k_. In the present sample, the resulting clusters contained 19, 26, and 7 items (cumulative variance explained by PC1 and PC2 of the global PCA: 78.8%).

**Step 5. Cluster-specific PCA and N-score extraction.** For each cluster *C*_k_, a cluster-specific PCA is performed on the sub-matrix **Z̃**_:,Ck_ containing only items belonging to that cluster. Let *w*_ki_ denote the first principal-component loading of item *i* within cluster *C*_k_. The N-score of participant *j* on cluster *k* is then defined as the weighted sum of the standardized item responses:

*N_kj_* = ∑_i ∈ Ck_ *w*_ki_ · z̃*_ij_*	(5)

Equation 5 yields three composite scores per participant—denoted *P_N1*, *P_N2*, and *P_N3* (collectively, the N-scores)—which were subsequently used as predictors in regularized logistic regression and random forest models with 10×5 repeated stratified cross-validation. Clinical validity of N-scores was assessed using Cohen’s *d* for ASD-vs-ND group differences and partial correlations with SRS-2 and VABS subscales controlling for age. The same pipeline was applied independently to the 153 Buddy Drill (D-item) accuracy vectors to derive D-item N-scores used in the hybrid Stage 2 analysis.
